# The effect of a brinzolamide/brimonidine fixed combination on optic nerve head blood flow in rabbits

**DOI:** 10.1371/journal.pone.0295122

**Published:** 2023-12-05

**Authors:** Nana Takahashi, Kota Sato, Naoki Kiyota, Mai Yamazaki, Eriko Kunikane, Toru Nakazawa

**Affiliations:** 1 Department of Ophthalmology, Tohoku University Graduate School of Medicine, Miyagi, Japan; 2 Department of Advanced Ophthalmic Medicine, Tohoku University Graduate School of Medicine, Miyagi, Japan; 3 Seiryo Eye Clinic, Miyagi, Japan; 4 Department of Research and Development Division, Senju Pharmaceutical Co., Ltd., Osaka, Japan; 5 Department of Ophthalmic Imaging and Information Analytics, Tohoku University Graduate School of Medicine, Miyagi, Japan; 6 Department of Retinal Disease Control, Tohoku University Graduate School of Medicine, Miyagi, Japan; University at Buffalo Jacobs School of Medicine and Biomedical Sciences: University at Buffalo School of Medicine and Biomedical Sciences, UNITED STATES

## Abstract

**Purpose:**

The purpose of this study was to investigate the effect of a 1% brinzolamide and 0.1% brimonidine fixed combination (BBFC) on ONH blood flow (BF) in rabbits.

**Methods:**

A crossover study was conducted on pigmented rabbits; a physiological saline solution, brinzolamide, or BBFC was administered for eight days. ONH BF, intraocular pressure (IOP) and systemic parameters were measured before the eighth day’s first dose and at 6, 9, 12, and 14 hours after the dose. ONH BF was assessed using laser speckle flowgraphy, and mean blur rate (MBR) values were calculated. The percentage against baseline of each parameter was calculated, and intergroup comparisons were performed at each time point.

**Results:**

There were no significant differences in the percentage change in systemic parameters. At 6 hours after administration, the BBFC group showed a significantly higher percentage change in large vessel area-MBR (%MV) compared to the control group (98.6±16.8%MV vs. 81.3±7.9%MV, P = 0.03). On the other hand, the brinzolamide group did not show a significant difference. Both the brinzolamide and BBFC groups had significantly lower percentage change in IOP (%IOP) compared to the control group (90.6±5.0%IOP, 93.3±2.9%IOP, and 99.2±1.7%IOP, respectively, P < 0.01).

**Conclusion:**

BBFC effectively reduces IOP and mitigates diurnal fluctuation-induced decreases in ONH BF.

## Introduction

Glaucoma, the second most common worldwide contributor to blindness [1], is defined by an ongoing decline in the number of retinal ganglion cells, leading to accompanying visual field abnormalities [2], While heightened intraocular pressure (IOP) remains the sole treatable risk factor for primary open-angle glaucoma (OAG) [3–5], the existence of normal-tension glaucoma underscores glaucoma’s multifactorial nature and implies that other elements beyond IOP can influence its advancement [6–9]. Vascular factors, including localized changes such as retinal arteriolar narrowing, are closely related to the progression of glaucomatous optic nerve damage caused by OAG [10–12]. Our previous studies using laser speckle flowgraphy (LSFG) have indicated that reduced optic nerve head blood flow (ONH BF) plays a significant role in the progression of anatomically corresponding visual field defects in eyes with OAG [13–15]. Additionally, we also found that this reduction in ONH BF can even precede structural changes represented by retinal nerve fiber layer thinning, at least in a subgroup of patients with OAG [16].

One of the limitations of these past studies were that they were primarily conducted in clinical settings with human subjects, leaving diurnal variations and nighttime changes in ocular BF largely unexplored. In fact, previous research has reported substantial nocturnal decline in ocular BF in eyes with primary OAG [17,18], suggesting that fluctuations in ONH BF, particularly nocturnal hypoperfusion, could potentially contribute to the development of glaucomatous optic neuropathy [18]. Thus, there is a demand for medications that can ameliorate this diurnal variation in ONH BF, including BF reductions at night.

Several studies have reported a positive effect on ocular BF after the administration of either brinzolamide [19–21] or brimonidine [22] eye drops. Therefore, we can expect that simultaneous administration of both eye drops could provide a greater protective effect on BF. However, an increase in the number of eye drops can result in issues with adherence in the real world [23]. Against this background, we used LSFG to investigate the diurnal variation in ONH BF in rabbit eyes and whether a brinzolamide and brimonidine fixed combination (BBFC) could inhibit the reduction of ONH BF due to diurnal variation.

## Materials and methods

### Animals

Ten male Dutch rabbits weighing 1.93 to 2.48 kilograms were obtained from Kitayama Labes Co, Ltd. (Nagano, Japan). Ten animals were used for the study of diurnal variation, and 9 of the same 10 animals were used for the study of the effectiveness of the eye drops. All rabbits were kept under controlled light/dark conditions, with food and water available ad libitum. The study was designed in accordance with the ARVO Statement Guidelines for the Use of Animals in Ophthalmic and Vision Research, and all rabbits were handled and all experiments were performed following the guidance of the Helsinki Declaration and with the approval of the Institutional Animal Care and Use Committee (IACUC) of our institution (approval number: #SJ21224).

### Experimental protocol for the evaluation of diurnal variation

Considering that diurnal variations in IOP and retinal BF have previously been reported [17,19,24–26], this study initially evaluated the temporal transition of IOP and ONH BF. Using normal, untreated rabbits, IOP and ONH BF were measured over time to confirm diurnal variation. The left eye was designated as the measurement eye; the measured parameters were IOP and ONH BF. Measurements were taken at 9:00, 15:00, 21:00, and 23:00. The measurements at 21:00 and 23:00 were performed under a red light.

### Experimental protocol for the evaluation of eye-drop effects

The study was conducted as a complete crossover trial, ensuring at least 7 days of washout for each animal to avoid any bias in the administration schedule. The rabbits were divided into groups, each receiving physiological saline solution, 1% brinzolamide (Novartis Pharma K.K., Tokyo, Japan) or a 1% brinzolamide and 0.1% brimonidine fixed combination (BBFC; Senju Pharmaceutical Co. Ltd, Osaka, Japan). Each treatment was administered twice daily, with the first instillation at 9:00 and the second under red light conditions 12 hours after the first instillation. This regimen was followed for 8 days, with repeated instillations in the left eye. On the 8th day of administration, IOP, ONH BF, blood pressure (BP), and pulse rate (PR) were measured before the first administration and 6, 9, 12, and 14 hours after the first administration.

### Measurement of IOP

The subject for IOP measurement was the left eye (the treated eye). We used a pneumatonometer (Model 30 Classic, Reichert Technologies) for this purpose. The animals were acclimatized to the restraint and the IOP testing environment for at least 3 days prior to the experiment. The animals were restrained in a restraint, and anesthetic eye drops **(**0.4% Benoxil; Santen Pharmaceutical Co. Ltd, Osaka, Japan) diluted 5 times with saline were applied twice (more than 5 minutes before and just before the measurement) to anesthetize the left eye. After the application of an eyelid speculum (Handaya Co. Ltd, Tokyo, Japan), IOP in the left eye was measured 3 times, and the average value was used for evaluation.

### LSFG measurements

This study used the LSFG-LITE device (Softcare Co., Ltd., Fukutsu, Japan) for evaluation of ONH BF. The principles of LSFG have previously been described in detail [27]. In essence, this equipment gauges the speckle contrast pattern arising when moving blood cells within vessels scatter laser interference. To accurately identify the ONH, we manually determined the measurement area of each rabbit’s ONH. Custom software then analyses the MBR map and divides it into areas characterized by the large vessels and tissue (i.e., capillaries) of the ONH; separate values can then be determined for the two areas. Vessel-area MBR (MV) has been reported to be closely correlated with measurements of BF in retinal vessels made with bidirectional laser Doppler velocimetry [28]. On the other hand, tissue-area MBR (MT) has been reported to be highly correlated with ONH BF in the capillaries measured using the hydrogen gas clearance technique and the microsphere method [29–31].

For ONH BF measurements, the left eye (ie, the eye that received the administration) was selected, and this assessment was conducted subsequent to the IOP measurement. The LSFG measurements were taken three times consecutively, and the average value of the three measurements was used for evaluation. For the LSFG imaging, the animals were secured using a restraint, as depicted in S1 Fig. Prior to the examination, the rabbits were acclimated to the restraint and the testing environment for the measurement of ONH BF for at least three days. Approximately 15 minutes before the measurement of ONH BF, 0.4% tropicamide (Mydrin M; Santen Pharmaceutical Co., Ltd, Osaka, Japan) was administered to dilate the pupils in the left eye. Also, an eyelid speculum was fitted, and a saline solution was administered as eye drops just before the start of the measurement to prevent drying of the eye during the measurement.

### Measurement of blood pressure and pulse rate

For the measurement of BP and PR, we used a BP100D II (Fukuda ME Kogyo Co., Ltd., Tokyo, Japan). Prior to the test, the animals were acclimated to the restraint and BP/PR measurement environment for at least 5 days. The BP/PR measurement was conducted following the LSFG measurement. The animals were restrained with a hanging-type restrainer, and a cuff was wrapped around the forelimb for approximately 5 minutes of quiet resting time. BP/PR measurements were taken 3 times; data measured during times when noise was minimal and the state of the animal was judged to be calm based on the waveform were identified, and the data closest to the measurement point was used.

Mean BP (MBP) and mean ocular perfusion pressure (MOPP) were calculated as follows: MBP = diastolic BP (DBP) + 1/3 (systolic BP [SBP]—DBP); MOPP = 2/3 MBP–IOP.

### Statistical analysis

All data are shown as the mean ± standard deviation (SD). The percentage against baseline of each parameter was calculated. An analysis of variance (ANOVA) and post-hoc Dunnett’s test were used to analyze the significance of differences in %MBP, %PR, %MOPP, %IOP, %MV, and %MT at each measurement point against the baseline of measurements at 9:00. A one-way ANOVA followed by the Tukey-Kramer test was used to analyze the significance of differences in %IOP, %MOPP, %MV, and %MT in the group comparisons of the evaluation of eye-drop effects at each time point. All statistical analyses were performed with JMP software (Pro version 16.1.0; SAS Institute Japan, Inc., Tokyo, Japan). The significance level was set at P < 0.05.

## Results

### Evaluation of diurnal variation

**Fig 1:**
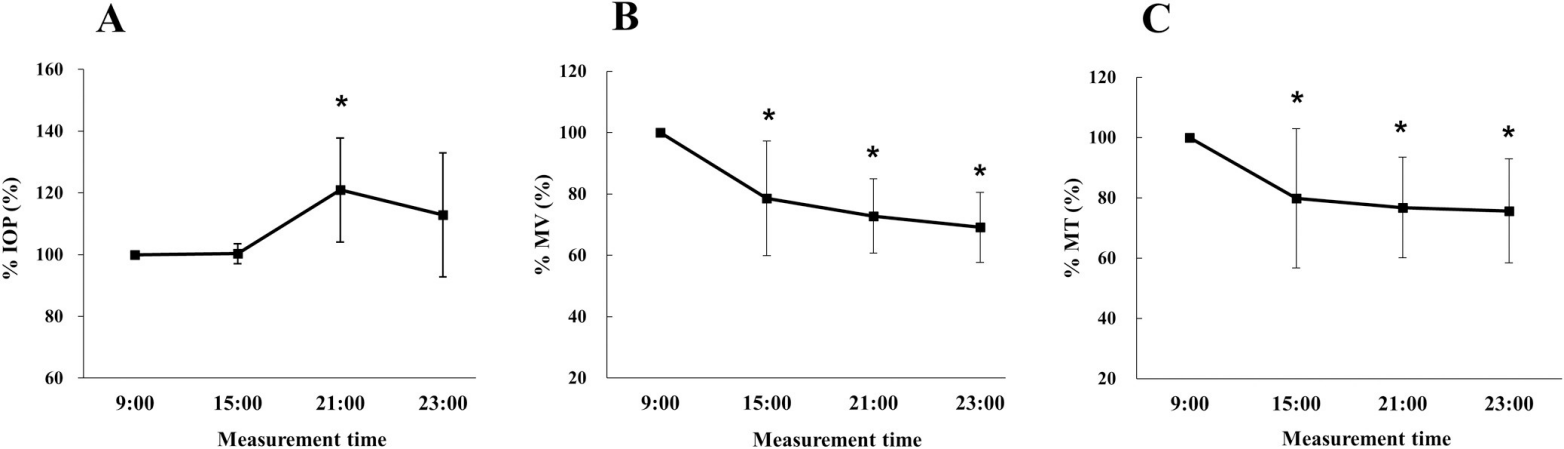
Diurnal changes in intraocular pressure and blood flow parameters in untreated rabbits. Diurnal variations in blood flow and intraocular pressure (IOP) as percentages in untreated rabbits (n = 10). The x-axis shows the measurement time, and the y-axis shows the percentage change in the indicated parameters. Measurements at 9:00 are taken as the baseline. Comparisons of %IOP (A), %MV (B), and %MT (C) at each measurement point were conducted using ANOVA and a post-hoc Dunnett’s test. Asterisks indicate significant differences (P < 0.05).

Firstly, we investigated the diurnal variation of IOP and ONH BF using normal, untreated rabbits. When using the 9:00 measurement as the baseline, the percentage against baseline at 15:00, 21:00, and 23:00 was, respectively, as follows: %IOP (100.3 ± 3.2%, P = 1.00; 120.9 ± 16.8%, P < 0.01; and 112.9 ± 20.0%, P = 0.09), %MV (78.6 ± 18.7%, P < 0.01; 72.8 ± 12.1%, P < 0.01; and 69.1 ± 11.4%, P < 0.01), and %MT (79.8 ± 23.1%, P = 0.03; 76.8 ± 16.7%, P < 0.01; and 75.7 ± 17.3%, P < 0.01). IOP exhibited diurnal variation, peaking at 21:00 (Fig 1A), whereas both MV and MT showed a peak at 9:00, followed by a decline until 23:00 (Fig 1B and 1C).

### Evaluation of eye-drop effects

**Fig 2:**
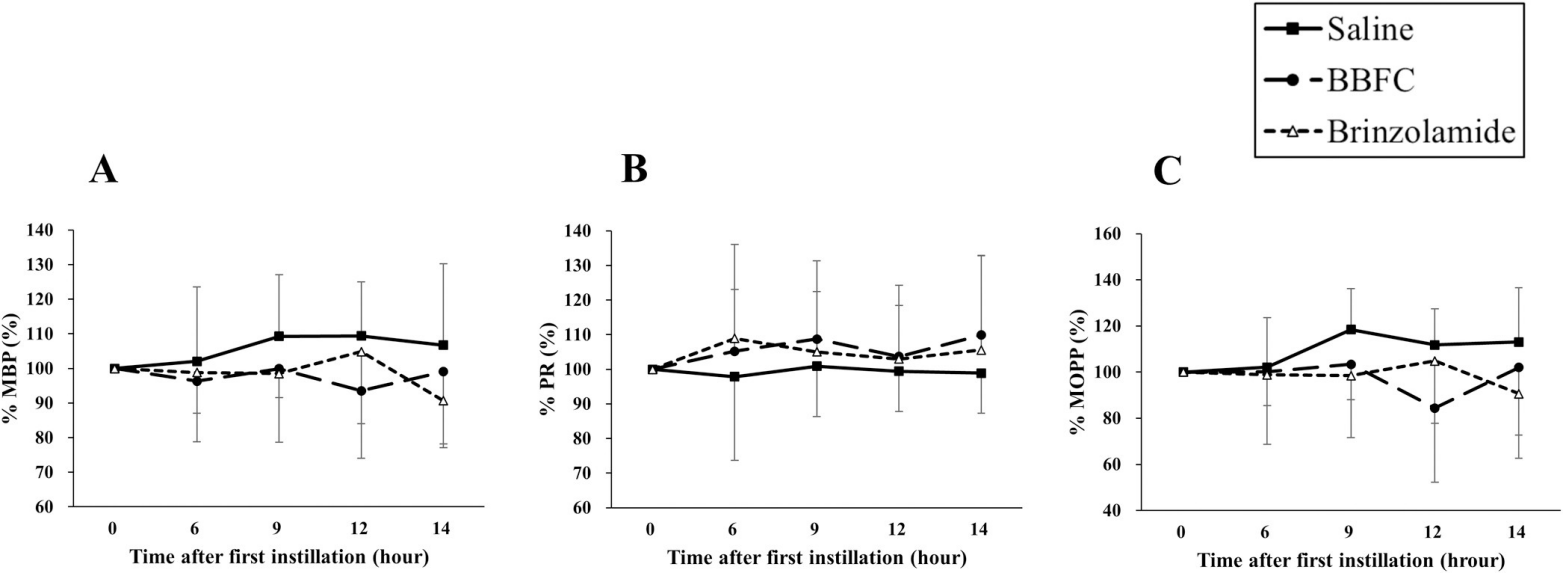
Evaluation of eye-drop effects on systemic parameters over time. Temporal changes as percentages for mean blood pressure (%MBP), pulse rate (%PR), and mean ocular perfusion pressure (%MOPP) during the evaluation of the effects of the eye drops. The x-axis shows the time after first instillation, and the y-axis shows %MBP (A), %PR (B), and %MOPP (C). The saline group, brinzolamide group, and brinzolamide and brimonidine fixed combination (BBFC) group are shown respectively by black squares (■), white triangles (△), and black circles (●). Each group had n = 9 and data are represented as mean±SD. A one-way ANOVA was used to compare groups.

**Fig 3:**
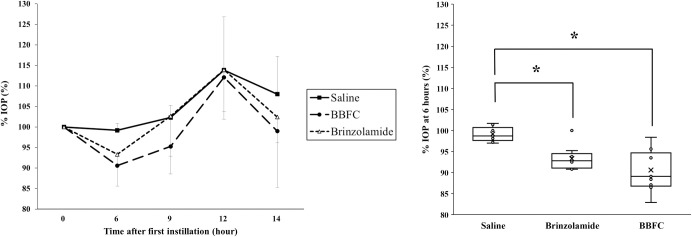
Temporal and intergroup analysis of intraocular pressure changes following eye drop administration. Changes in intraocular pressure (IOP) as percentages (%IOP) during the evaluation of the effects of the eye drops. The left figure shows temporal changes in %IOP in the saline group, brinzolamide group, and brinzolamide and brimonidine fixed combination (BBFC) group, respectively, shown by black squares (■), white triangles (△), and black circles (●). The right figure shows an intergroup comparison of %IOP 6 hours after administration. Each group had n = 9. Error bars designate SD. *P < 0.05 in a one-way ANOVA followed by the Tukey-Kramer test.

At all measurement points, no significant differences were observed in the comparisons among groups for %MBP, %PR, and %MOPP (P = 0.22–0.87; ANOVA) (Fig 2). Six hours after administration, the BBFC-treated group and the brinzolamide monotherapy group showed significantly lower %IOP compared to the control group (90.6 ± 5.0%, 93.3 ± 2.9%, and 99.2 ± 1.7%, respectively; P < 0.01) (Fig 3). At 9, 12, and 14 hours after administration, the BBFC-treated group tended to have lower %IOP compared to the brinzolamide monotherapy group and the control group, but these differences were not statistically significant (95.3 ± 6.7%, 102.3 ± 3.0%, and 102.7 ± 9.8%, respectively; P = 0.06; 112.1 ± 10.2%, 113.9 ± 10.1%, and 113.9 ± 13.0%, respectively; P = 0.93; 199.0 ± 13.7%, 102.4 ± 6.3%, and 108.0 ± 9.2%, respectively; P = 0.19; ANOVA).

**Fig 4:**
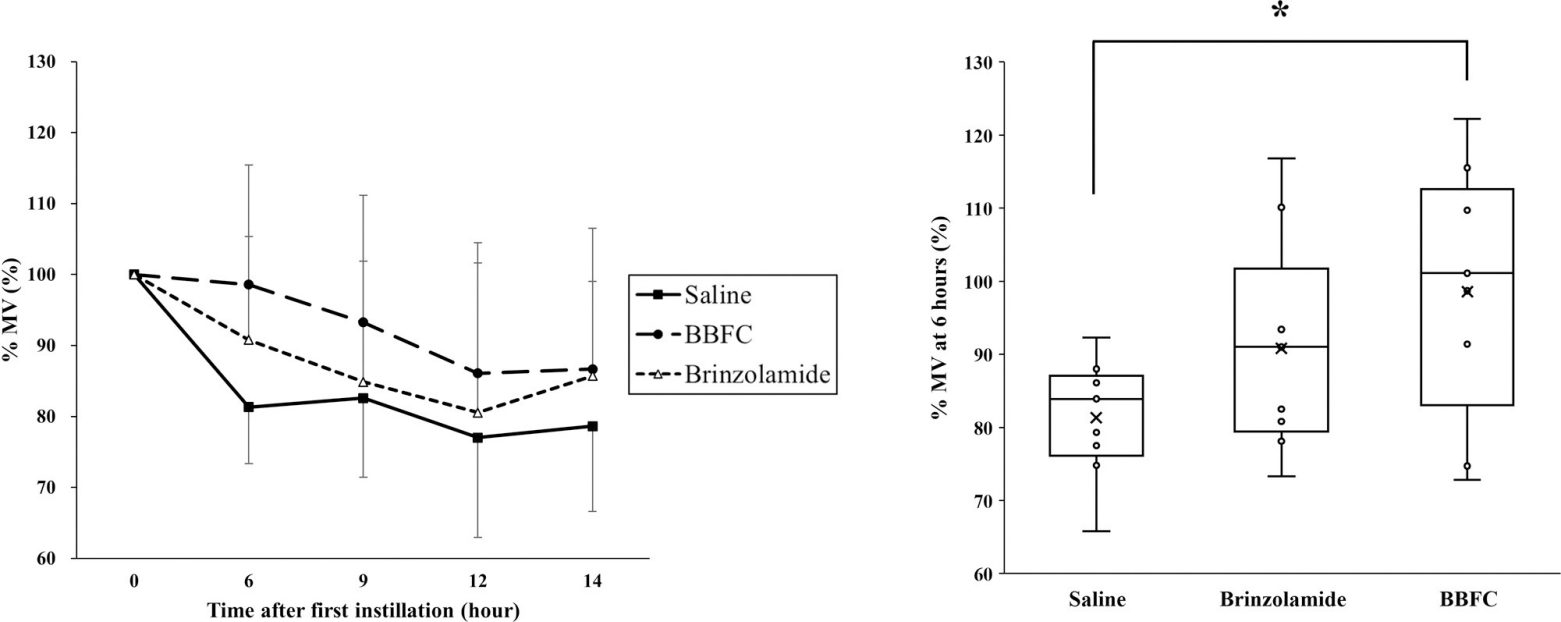
Temporal change and intergroup comparison of vessel area mean blur rate following eye drop administration. Changes in vessel area mean blur rate as percentages (%MV) during the evaluation of the effects of the eye drops. The left figure shows temporal changes in %MV in the saline group, brinzolamide group, and brinzolamide and brimonidine fixed combination (BBFC) group, respectively, shown by black squares (■), white triangles (△), and black circles (●). The right figure shows intergroup comparisons of %MV 6 hours after administration. Each group had n = 9. Error bars designate SD. *P < 0.05 in a one-way ANOVA followed by the Tukey-Kramer test.

Six hours after administration, the BBFC-treated group showed significantly higher %MV compared to the control group (98.6 ± 16.8% vs 81.3 ± 7.9%, P = 0.03) (Fig 4), while there was no significant difference between the brinzolamide monotherapy group and the control group (90.8 ± 14.5% vs 81.3 ± 7.9%, P = 0.32) (Fig 4). At 9, 12, and 14 hours after administration, the BBFC-treated group tended to have higher %MV compared to the brinzolamide monotherapy group and the control group, but these differences were not statistically significant (93.3 ± 17.9%, 84.9 ± 17.0%, and 82.6 ± 11.1%, respectively; P = 0.33; 86.1 ± 18.4%, 80.6 ± 21.1%, and 77.0 ± 14.1%, respectively; P = 0.57; 86.7 ± 19.9%, 85.7 ± 13.3%, and 78.6 ± 12.0%, respectively; P = 0.49, ANOVA).

**Fig 5:**
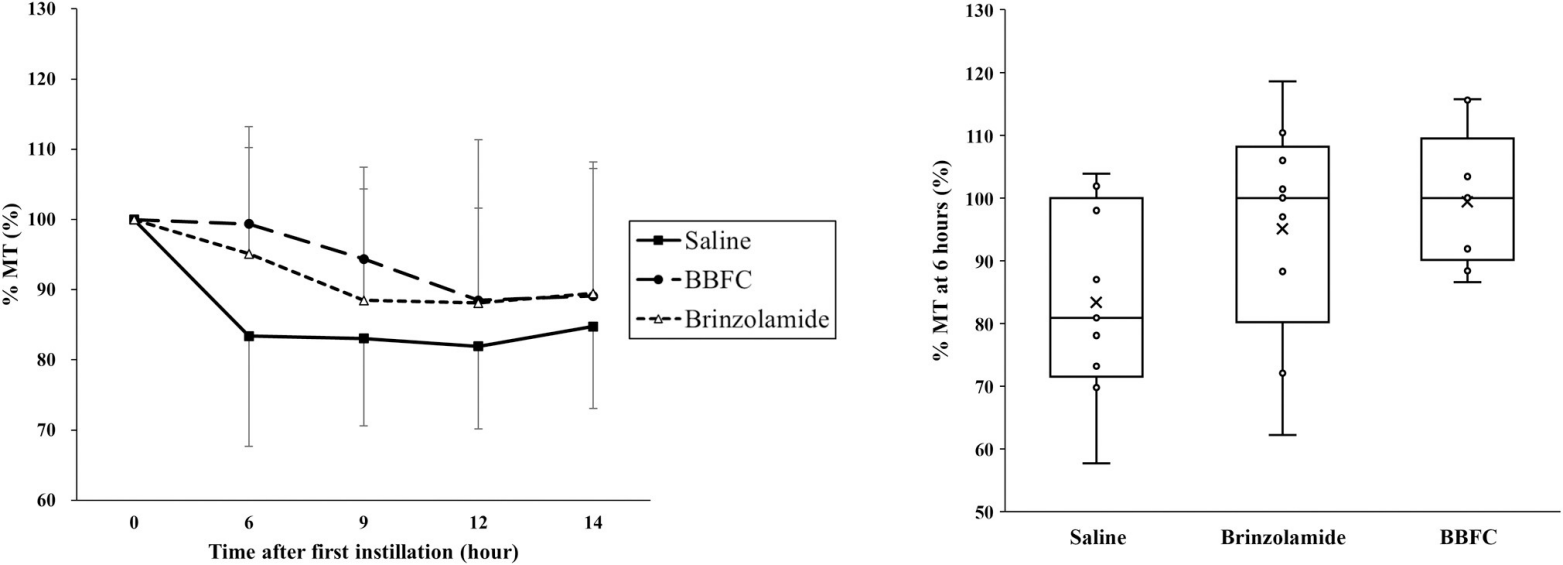
Temporal change and intergroup comparison of tissue area mean blur rate following eye drop administration. Changes in tissue area mean blur rate as percentages (%MT) during the evaluation of the effects of the eye drops. The left figure shows temporal changes in %MT in the saline group, brinzolamide group, and brinzolamide and brimonidine fixed combination (BBFC) group, respectively, shown by black squares (■), white triangles (△), and black circles (●). The right figure shows intergroup comparisons of %MT 6 hours after administration. Each group had n = 9. Error bars designate SD. A one-way ANOVA was used for group comparisons.

At 6, 9, 12, and 14 hours after administration, both the BBFC-treated group and the brinzolamide monotherapy group tended to have higher %MT compared to the control group, but these differences were not statistically significant (99.4 ± 10.9%, 95.1 ± 18.1%, and 83.4 ± 15.7%, respectively; P = 0.09; 94.4 ± 13.1%, 88.5 ± 15.9%, and 83.1 ± 12.5%, respectively; P = 0.24; 88.5 ± 13.1%, 88.1 ± 23.2%, and 81.9 ± 11.8%, respectively; P = 0.66; 89.1 ± 18.1%, 89.5 ± 18.7%, and 84.7 ± 11.7%, respectively; P = 0.80; ANOVA) (Fig 5).

**Fig 6:**
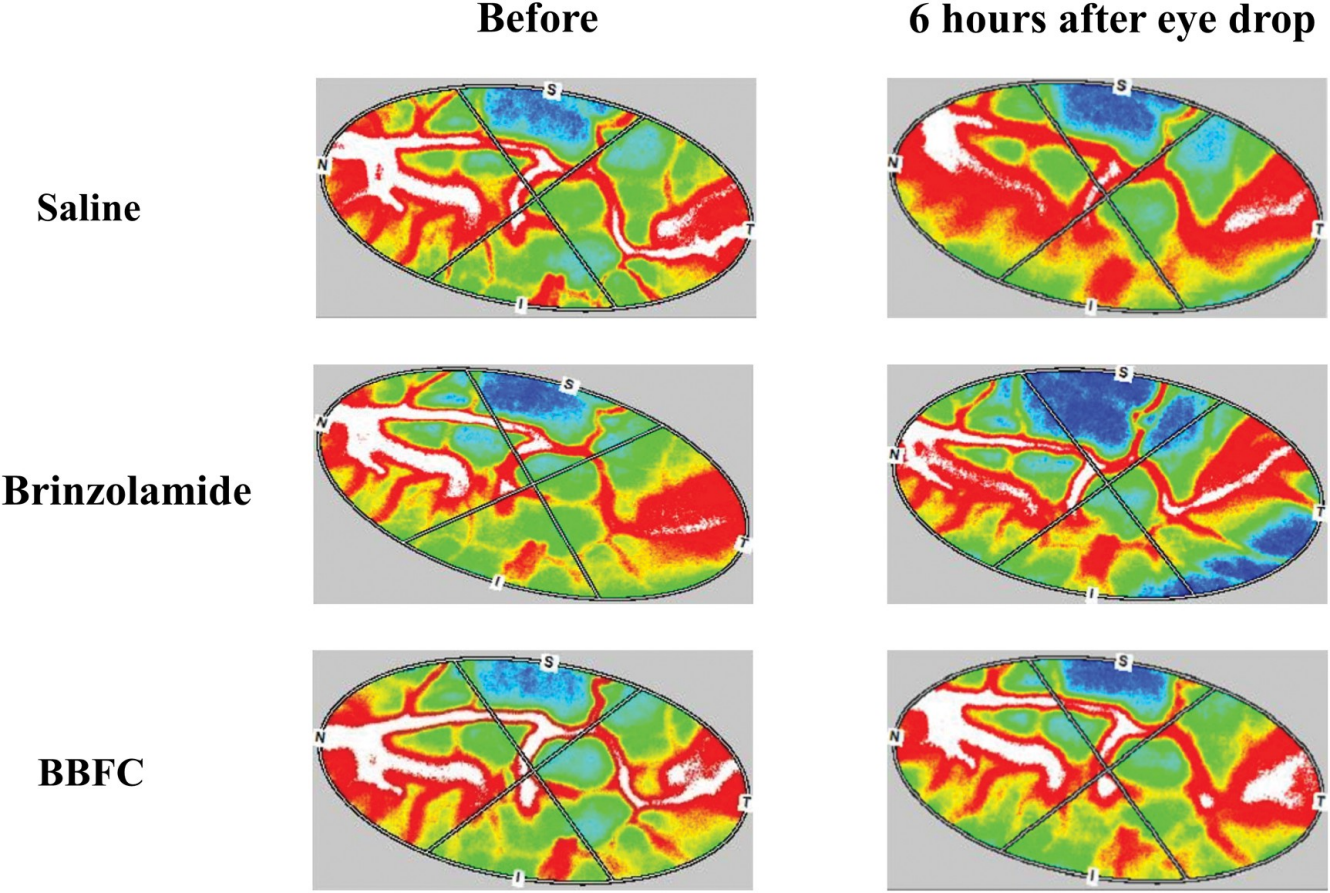
Changes in optic nerve head blood flow following eye drop administration in a representative case. This figure shows the changes in optic nerve head blood flow before and 6 hours after eye drop administration in a representative case from each group. The images are color maps captured using laser speckle flowgraphy. In the brinzolamide and brimonidine fixed combination (BBFC) group, a suppression of diurnal fluctuation-induced decreases in optic nerve head blood flow was observed 6 hours after administration compared to the baseline.

Fig 6 shows the changes in ONH BF before and after eye drop administration in a representative case.

After administering BBFC to pigmented rabbits twice daily at 12-hour intervals for 8 days, lower %IOP and higher %MV were observed 6 hours after administration compared to the brinzolamide group and the control group.

## Discussion

In this study, we examined diurnal variations in IOP and ONH BF and investigated the effect of BBFC on these parameters in rabbit eyes. Untreated healthy rabbits exhibited a higher nocturnal IOP at 21:00 compared to diurnal IOP, while both MV and MT peaked at 9:00 in the morning and decreased gradually until 23:00. Six hours after BBFC administration, a significant reduction in IOP and an amelioration of MV decline, i.e., diurnal fluctuation, were observed, while there were no significant intergroup differences in MBP, MOPP, or PR throughout the day.

The diurnal fluctuations in IOP and ONH BF that we observed in this study are consistent with previous studies, which have reported that IOP is higher at night in both healthy individuals and patients with glaucoma [19,24–26,32,33]. While this does not contradict our findings, it is difficult to directly compare our study and prior ones because humans change their body position, especially during sleep, from the sitting to supine position. On the other hand, Ya-Ru et al. observed diurnal fluctuations in rabbits that were similar to those of humans and found that IOP peaked at 21:00 and ONH BF peaked at 9:00 in the morning and then decreased [19], findings that are consistent with ours. Thus, it can be said that our experiments are reproducible and reliable.

On the other hand, we did not observe any differences in %MBP, %PR, and %MOPP between the control, brinzolamide, and BBFC groups. There have been discussions concerning the BP-lowering effect of α2 stimulators [34], a component of BBFC, while on the other hand, α2 stimulators have been clinically demonstrated to be safe and to not exert harmful effects on cardiovascular parameters [35–37], which seems to be consistent with our findings. In our results, %MOPP at 6 hours after administration was 108.8 ± 50.4% in the control group, 103.7 ± 32.1% in the brinzolamide group, and 100.2 ± 15.7% in the BBFC group. These values are all within the reported range of autoregulatory capacity of ocular BF (i.e., around 40–175%) [38–42]. Given that there were no intergroup differences in vital signs and that %MOPP was within the range of autoregulation, the effect of ONH BF change observed in our study was likely due to a local pharmacological effect of BBFC other than the IOP-lowering effect.

Interestingly, we observed a significant increase in %MV 6 hours after administering BBFC, while brinzolamide alone and %MT failed to produce statistically significant changes, though the trend was similar. The stronger BF improvement effect of BBFC compared to brinzolamide alone suggests the presence of a BF-improving effect in brimonidine as an α2 stimulator [22,43–45]. While α2 agonists have been reported to cause vasoconstriction in some capillaries [46–48], they have been shown to cause vasodilation in other, larger vessels [47,49,50]. In the eye, it has been reported that BBFC eye drops reach the vitreous body and retinal vessels [51]. When the vitreous concentration of brimonidine surpasses the threshold for α2 adrenergic receptor activation [52], the expression of endothelial nitric oxide synthase (eNOS) increases, inducing NO, which has a vasodilating effect on the vasculature, including in the retinal vessels [53]. Therefore, the BF-improving effect of BBFC observed in this study is valid. On the other hand, while there was a tendency for an increase in MT, the lack of statistical significance could be attributed to several possibilities. One is that the MT value (i.e., capillary BF) value was smaller than the MV value (i.e., large vessel BF), making it difficult to capture changes. Another possibility is that, as described, the effect of α2 agonists can vary between constriction and dilation depending on vasculature size, so the vasodilating effect could actually be lower in the ONH capillaries. In fact, Rosa RH et al. observed that eNOS expression and the BF-improving effect of brimonidine were less prominent in the capillaries than larger vessels in a pig-retina ex vivo model. [53] More detailed research is necessary to elaborate this point.

In this study, both IOP and ONH BF increased only at 6 hours after the first administration of BBFC, while the effects were not evident at other time points, such as 9, 12, and 14 hours after the first instillation. According to previous reports, the peak impact of brimonidine and brinzolamide on IOP or ocular BF is reported to occur within a 3–9-hour range [19,22,54,55], which is fundamentally consistent with our findings. Although the eye drops were administered twice a day in this study, with the second instillation given 12 hours after the first, it is reasonable that effects on IOP and ocular BF were not observed at the 12- and 14-hour time points. The cause for the lack of significant ONH BF change 9 hours after the first instillation remains unclear. Further research with a more detailed time course is warranted.

Our study has several limitations. First, we used normal rabbits; extrapolating the results from this rabbit model directly to human patients with glaucoma might not be straightforward. Second, LSFG cannot directly provide data on blood flow velocity, and the depth at which it measures blood flow remains unclear. Further investigation is needed to clarify these points in the future.

In conclusion, this study demonstrated the effects of BBFC in reducing IOP and ameliorating the reduction of ONH BF due to diurnal variation 6 hours after the first instillation. Therefore, BBFC may represent a valuable therapeutic option for glaucoma in terms not only of IOP reduction but also ONH BF change.

## Supporting information

S1 Data All relevant data. All relevant data are available in S1 Data.
(XLSX)

S1 Fig The methods of restraint to secure the animal during measurements. Picture showing the use of a restraint to secure the animal during intraocular pressure measurement and laser speckle flowgraphy imaging.
(TIF)
