# Supplementary figures and images for: The effect of a brinzolamide/brimonidine fixed combination on optic nerve head blood flow in rabbits
Source: PLoS One. 2023 Dec 5;18(12):e0295122. doi: 10.1371/journal.pone.0295122 (PMC10697578; doi:10.1371/journal.pone.0295122)

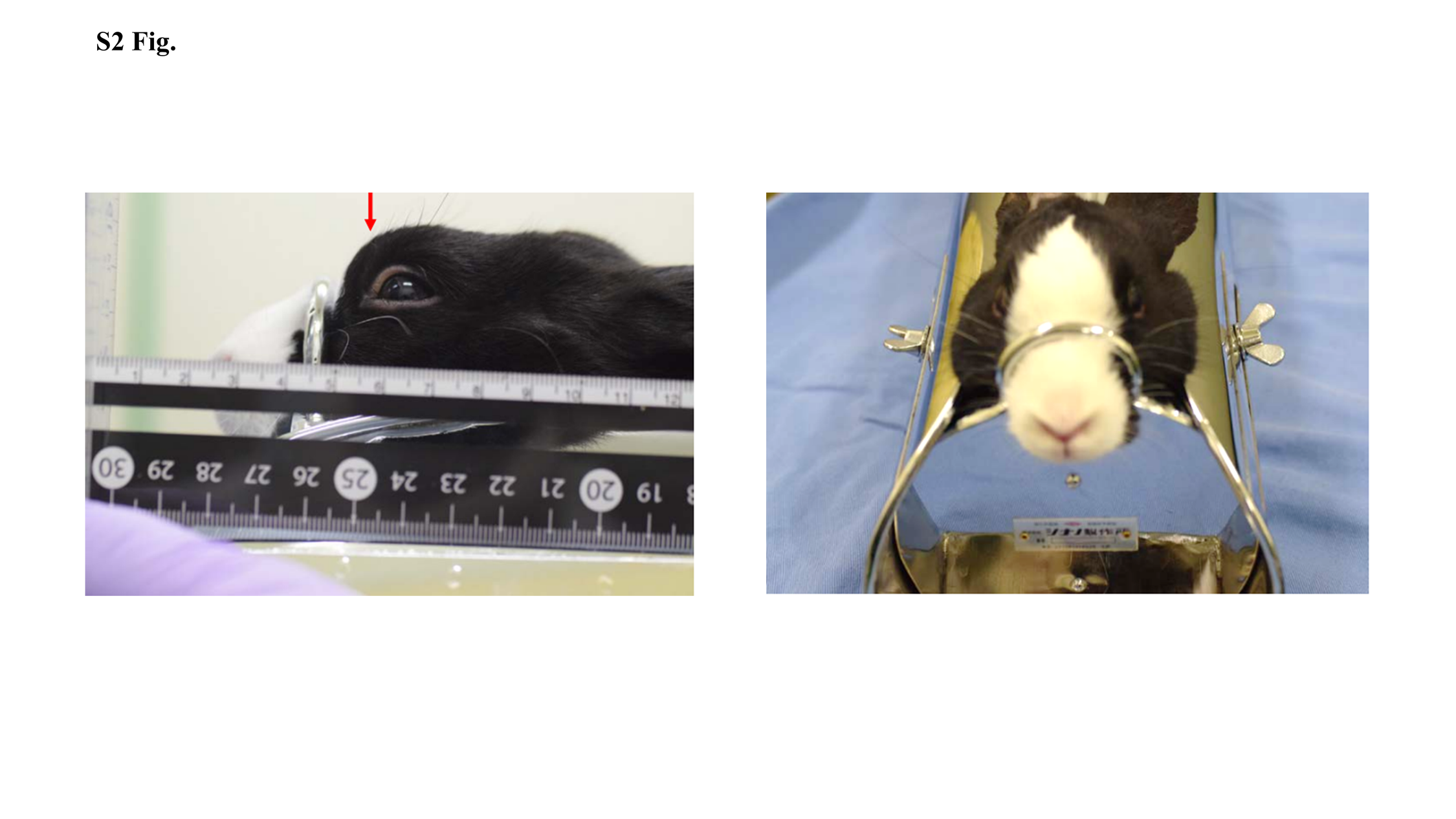

Supplement: S1 Fig — Picture showing the use of a restraint to secure the animal during intraocular pressure measurement and laser speckle flowgraphy imaging. (TIF) [file pone.0295122.s002.tif]
